# PP2A inhibition results in hepatic insulin resistance despite Akt2 activation

**DOI:** 10.18632/aging.100611

**Published:** 2013-10-21

**Authors:** Thomas Galbo, Rachel J. Perry, Erica Nishimura, Varman T. Samuel, Bjørn Quistorff, Gerald I. Shulman

**Affiliations:** ^1^ Department of Internal Medicine, Yale University School of Medicine, New Haven, CT 06510, USA; ^2^ Department of Cellular & Molecular Physiology, Yale University School of Medicine, New Haven, CT 06510, USA; ^3^ Department of Insulin Biology, Diabetes Research Unit, Novo Nordisk, Måløv, Denmark; ^4^ Department of Biomedical Sciences, University of Copenhagen, Copenhagen, Denmark; ^5^ VA Medical Center, West Haven, CT 06516, USA; ^6^ Howard Hughes Medical Institute, New Haven, CT 06516, USA; ^7^ Novo Nordisk Center for Basic Metabolic Research, Copenhagen, Denmark

## Abstract

In the liver, insulin suppresses hepatic gluconeogenesis by activating Akt, which inactivates the key gluconeogenic transcription factor FoxO1 (Forkhead Box O1). Recent studies have implicated hyperactivity of the Akt phosphatase Protein Phosphatase 2A (PP2A) and impaired Akt signaling as a molecular defect underlying insulin resistance. We therefore hypothesized that PP2A inhibition would enhance insulin-stimulated Akt activity and decrease glucose production. PP2A inhibitors increased hepatic Akt phosphorylation and inhibited FoxO1 *in vitro* and *in vivo*, and suppressed gluconeogenesis in hepatocytes. Paradoxically, PP2A inhibition exacerbated insulin resistance *in vivo*. This was explained by phosphorylation of both hepatic glycogen synthase (GS) (inactivation) and phosphorylase (activation) resulting in impairment of glycogen storage. Our findings underline the significance of GS and Phosphorylase as hepatic PP2A substrates and importance of glycogen metabolism in acute plasma glucose regulation.

## INTRODUCTION

Hepatic insulin resistance is a defining feature of type 2 diabetes (T2D) but the cellular and molecular mechanisms responsible for this insulin resistance remain unknown [1]. Insulin normally acts to suppress hepatic glucose production (HGP) by inhibiting gluconeogenesis and stimulating net glycogen synthesis, however, this ability is impaired in insulin resistance and T2D [2-4].

Akt is a key signaling node in hepatic insulin action [5] and especially the Akt2 isoform appears to be critical for insulin action on glucose metabolism *in vivo* [6]. Insulin stimulates phosphorylation and activation of Akt that in turn acts to phosphorylate and inactivate the transcription factor FoxO1, which induces transcription of the gluconeogenic enzymes glucose-6-phosphatase (G6pc) and phosphoenolpyruvate carboxykinase 1 (Pepck) and enhances gluconeogenesis under fasting conditions [7-9]. Similarly, insulin-stimulated Akt activation also leads to phosphorylation and inactivation of Gsk3α [10]. Since Gsk3α normally phosphorylates and inhibits GS, this results in increased GS activity and glycogen synthesis.

While Akt is dependent on stimulatory signals from the insulin receptor (IR) for activation it is simultaneously under negative control by phosphatases [11] most importantly PP2A [12]. PP2A controls the phosphorylation level of a wide range of substrates and is believed to gain specificity through utilization of a plethora of particular targeting regulatory subunits [13]. Recent studies have implicated saturated fatty acid-induced hyperactivity of PP2A in the pathogenesis of insulin resistance, at the level of Akt activation, in all major insulin responsive cell types [14-18].

Using small molecule inhibitors such as cantharidin and LB1 it is possible to manipulate PP2A activity. The natural toxin cantharidin inhibits PP2A *in vitro* [19, 20] while the non-toxic norcantharidin (demethylated cantharidin)-analog LB1 is highly specific for PP2A and suitable for inhibition of PP2A *in vivo* [21].

As previous work had indicated that PP2A inhibition could rescue hepatic Akt activity in insulin resistant states [16] we hypothesized that PP2A inhibition in the liver would lead to increased insulin-stimulated inhibition of FoxO1, thus potentially having therapeutic applications in T2D. To address these questions we performed studies in primary rat hepatocytes as well as in rats fed either a chow diet or a three day high-fat diet, a well-established model of hepatic insulin resistance [22].

## RESULTS

### Fatty acids increase hepatic PP2A activity in vitro and in vivo and small molecule inhibitors can be used to impair hepatic PP2A activity

**Figure 1:**
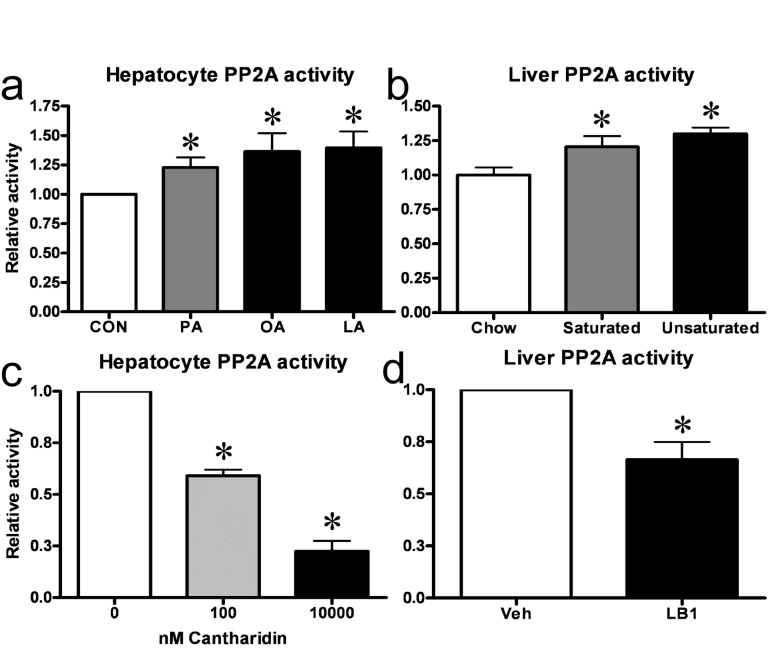
Fat increases hepatic PP2A *in vitro* and *in vivo* and small molecule inhibitors can be used to impair hepatic PP2A activity. Treatment of primary hepatocytes with either of palmitate (PA), oleate (OA) or linoleate (LA) resulted in an increase in PP2A activity **(a)**. Similarly, three day fat-feeding with a diet based on either saturated or unsaturated fats led to an increase in hepatic PP2A activity in rats **(b)**. 30 mins of cantharidin treatment resulted in a dose-dependent inhibition of PP2A-activity in primary rat hepatocytes **(c)** while 3 hrs of LB1-treatment led to inhibition of PP2A in rat livers **(d)**. Relative activity is relative to no treatment. Data are averages of PP2A activity assays ±SEM. * P<0.05.

We first sought to establish whether PP2A activity was altered in states of hepatic insulin resistance. As exposure to fatty acids is a model system of insulin resistance *in* vitro and *in vivo* we measured PP2A activity in primary rat hepatocytes cultured with 0.5mM of either of the fatty acids palmitate, oleate or linoleate as well as in livers from rats fed a three day high-fat diet based on either saturated or unsaturated fat. We found that exposure to any of the fatty acids resulted in a 20-25% increase in hepatocyte PP2A activity (Figure 1a) and that feeding both saturated and unsaturated fat-based diets similarly increased hepatic PP2A activity in rats *in vivo* (Figure 1b). To study the function of hepatic PP2A activity, we utilized the small molecule inhibitors P2A activity asin ither palmitate, oleate or linoleateed fatcantharidin and LB1, which inhibited PP2A activity in our *in vitro* and *in vivo* model systems respectively. In hepatocytes, 30 mins of treatment with cantharidin resulted in inhibition of PP2A activity (Figure 1c) with an approximately 75% inhibition of PP2A activity being observed in cells given 10uM cantharidin. In rats, we found that intraperitoneal injection of 2mg/kg LB1 resulted in inhibition of hepatic PP2A activity with a maximal effect of 35% inhibition observed 3 hours post injection (Figure 1d).

### PP2A inhibition leads to activation of insulin-like signaling, suppression of gluconeogenic gene expression and gluconeogenesis in primary hepatocytes

**Figure 2:**
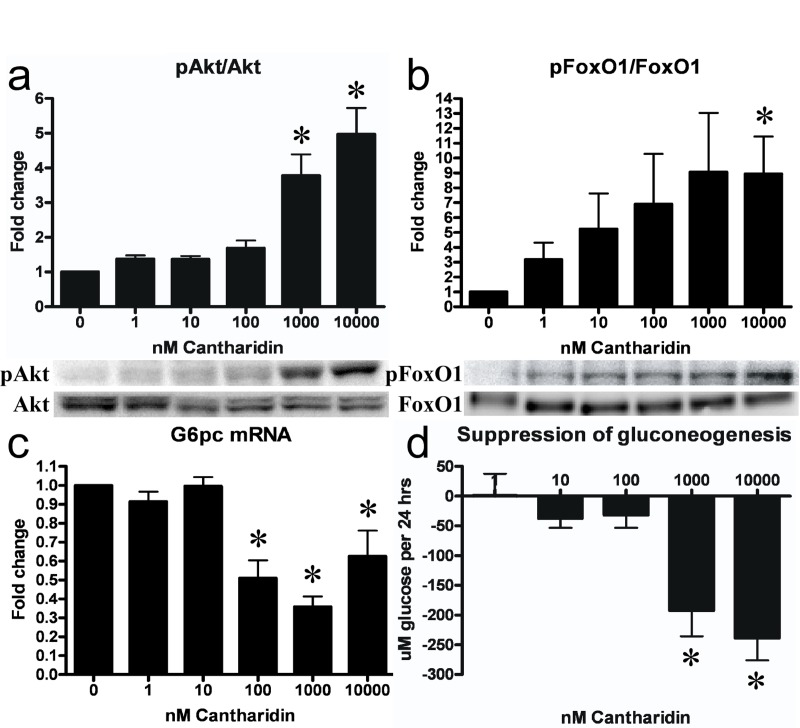
PP2A inhibition leads to activation of insulin signaling and ensuing suppression of gluconeogenic gene transcription and gluconeo-genesis in hepatocytes. Cantharidin-treatment led to a dose-dependent increase in phosphorylation (Ser473) and activation of Akt **(a)**. This effect was conferred onto the Akt substrate FoxO1 (Ser256), which was phosphorylated and inactivated. The inactivation of FoxO1 led to a decreased transcription of the gluconeogenic gene G6pc **(c)** and a reduction in the rate of gluconeogenesis **(d)**. Fold change is relative to no treatment. Data are averages of western blot quantifications, real-time PCR results and gluconeogenesis assays ±SEM. * indicates p<0.05. Representative western blots are shown.

As PP2A is a negative regulator of Akt phosphorylation and activation, we assessed the effect of PP2A inhibition upon Akt-dependent insulin signaling in hepatocytes. Treatment with cantharidin for 30 mins did not affect IRβ tyrosine phosphorylation and activation (Figure S1), but resulted in a dose-dependent activation of Akt (Figure 2a). Cantharidin-induced activation of Akt was intact in the presence of the potent IR inhibitor S961 [23] (not shown) confirming that the effects of cantharidin were independent of IR activation. This effect was propagated onto the Akt substrates Gsk3α and FoxO1 (Figure S2 and Figure 2b), which were phosphorylated and inactivated in response to Akt activation. In cells given 10uM cantharidin we observed a 40-50% downregulation of total FoxO1 protein level (Figure S3) similar to what is observed in hepatocytes under conditions of intense insulin-stimulated Akt signaling [9, 24]. As FoxO1 is a key gluconeogenic transcription factor that drives G6pc and Pepck mRNA transcription we examined the effect of PP2A inhibition on the expression of these enzymes and gluconeo-genesis. After 2 hrs of cantharidin-treatment, both G6pc and Pepck mRNA were downregulated in hepatocytes (Figure 2c and Figure S4) resulting in suppression of hepatocyte gluconeogenesis (Figure 2d).

### Acute in vivo PP2A inhibition improves acute hepatic insulin-like signaling in fat-fed rats

**Figure 3:**
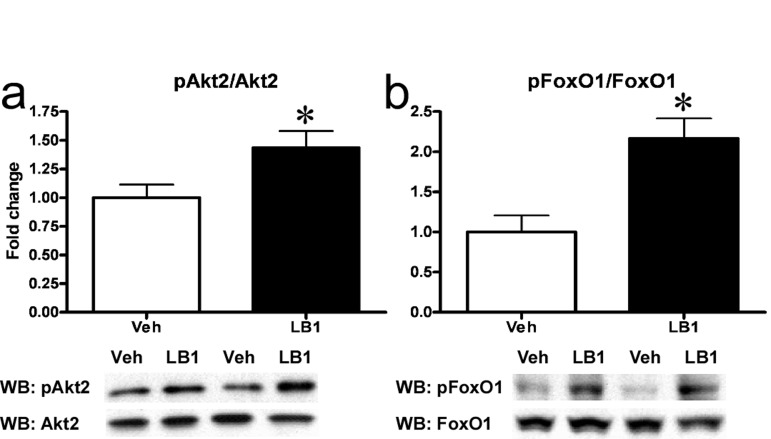
PP2A inhibition acutely improves hepatic insulin signaling in fat-fed rats. I.p.-injection of LB1 resulted in an augmented insulin-stimulated phosphorylation (Ser474) and activation of hepatic Akt2 (Ser474) **(a)** and phosphorylation (Ser256) and inactivation of FoxO1 **(b)** *in vivo*. Fold change is relative to insulin-stimulated, vehicle-injected rats. Data are averages of western blot quantifications ±SEM. * P<0.05.

Following our *in vitro* observations, we sought to determine whether inhibition of PP2A would enhance hepatic insulin signaling *in vivo* in a model of hepatic insulin resistance. To test this, we treated rats with LB1 and assessed Akt2-FoxO1 signaling. LB1-treatment resulted in a 25-30% and 75% increase in basal phosphorylation of Akt2 and FoxO1 (not shown) in fat-fed rats, while enhancing insulin-stimulated Akt2 activation by ~35% (Figure 3a) and resulting in a more than two-fold increase in insulin-stimulated phosphorylation of FoxO1 (Figure 3b) compared to vehicle, in rats infused with insulin.

### Acute in vivo PP2A inhibition exacerbates insulin resistance in chow- and fat-fed rats

**Figure 4:**
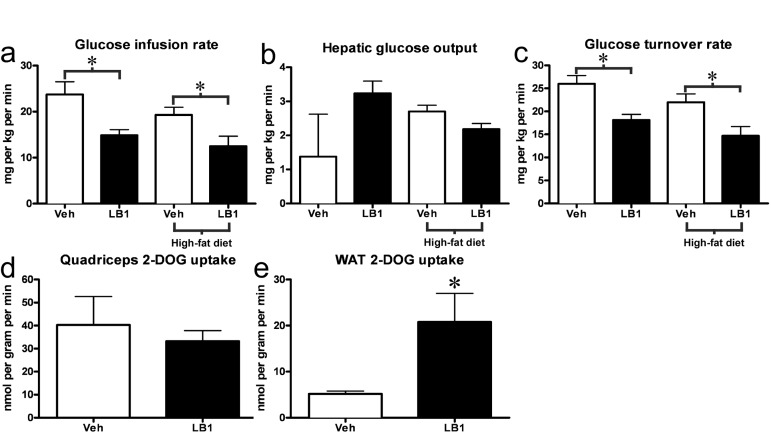
PP2A inhibition acutely exacerbates insulin resistance in chow- and fat-fed rats. LB1-treated rats required a reduced glucose infusion rate to maintain euglycemia during the hyperinsulinemic-euglycemic clamp **(a)**. This was not associated with changes in hepatic glucose output **(b)**, but was the result of an impaired glucose turnover rate **(c)**. Muscle insulin sensitivity was not altered as assessed by 2-deoxyglucose uptake (2-DOG) (**d**), however LB1-treatment was associated with an increase in insulin-stimulated glucose uptake in white adipose tissue (**e**). Data are averages ±SEM. * P<0.05.

Having seen that LB1-treatment improved acute hepatic insulin signaling we hypothesized that PP2A inhibition would result in increased insulin sensitivity in chow- and fat-fed rats. To address this question, we inhibited PP2A using LB1 as previously, before submitting them to a hyperinsulinemic-euglycemic clamp. As we had observed in our previous experiments, LB1-treatment led to a ~30-40% increase in insulin-stimulated hepatic activation of Akt2 (not shown) and a more than two-fold increase in phosphorylation and inactivation of FoxO1 (not shown). However, LB1-treated rats displayed a 8-10 mg/dL increase in fasting plasma glucose levels compared to vehicle-treated rats and were significantly more insulin resistant as reflected by the ~30% reduction in glucose infusion rate required to maintain euglycemia during the hyperinsulinemic clamp (Figure 4a). PP2A inhibition was not associated with a change in hepatic glucose output (Figure 4b). Rather, glucose disposal was impaired as LB1-treatment resulted in a ~25-30% decrease in whole body glucose turnover rate determined by stable isotope infusion (Figure 4c) accounting for the decreased glucose infusion rate. Muscle insulin sensitivity, as determined by 2-deoxyglucose (2-DOG) uptake, was not different in LB1-treated chow-fed rats (Figure 4d), however, LB1-treatment was associated with an increase in insulin-stimulated white adipose tissue (WAT) glucose uptake (Figure 4e).

### Acutely enhanced hepatic signaling through the Akt-FoxO1 node is not associated with decreased G6Pase and Pepck protein levels

**Figure 5:**
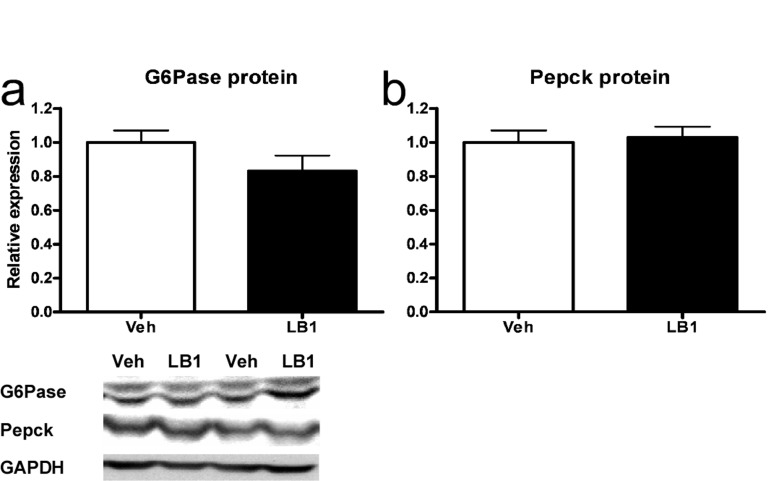
Acutely increased insulin-stimulated signaling through the Akt-FoxO1 node following PP2A inhibition does not result in reduced G6Pase and Pepck protein levels in rat livers. LB1-treatment did not lead to decreased levels of G6Pase **(a)** nor Pepck **(b)** protein despite PP2A inhibition resulting in inactivation of FoxO1. Data are averages of western blot quantifications ±SEM. * P<0.05. Representative western blots are shown.

The main effects of hepatic insulin action on glucose metabolism are to suppress gluconeogenic transcription, through the Akt2-FoxO1 node, and simultaneously to promote net glycogen synthesis. Our studies had established that PP2A-inhibition resulted in increased signaling through the Akt2-FoxO1 node as well as decreased levels of G6pc and Pepck mRNA. We therefore sought to determine whether the protein levels of these enzymes were reduced in the livers of clamped animals treated with LB1. However, despite the robust increase in FoxO1 phosphorylation, there was only a weak tendency toward lowered G6Pase (Figure 5a) while Pepck protein levels were not different in the post-clamp livers of rats treated with the PP2A-inhibitor (Figure 5b).

### PP2A inhibition results in inhibition of signaling promoting nutrient storage

**Figure 6:**
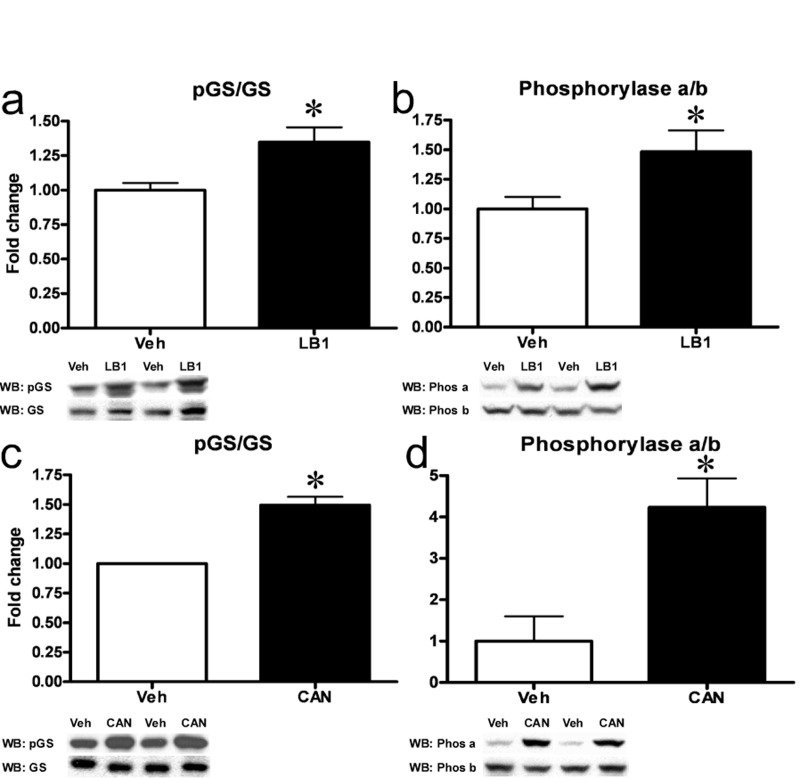
Inhibition of hepatic PP2A results in inactivation of GS and activation of Phosphorylase. LB1-treatment resulted in increased phosphorylation (Ser641) and inactivation of GS **(a)** while increasing the ratio of active Phosphorylase a to inactive b **(b)** in the livers of fat-fed rats. Cantharidin-treatment in primary rat hepatocytes mimicked these effects and resulted in inactivation of GS **(c)** and activation of Phosphorylase **(d)**. Data are averages of western blot quantifications ±SEM. * P<0.05. Representative western blots are shown.

Recent attention drawn by PP2A, in the context of insulin signaling and metabolic disease, has been focused on the inhibitory effects elicited on Akt. However, while PP1 is the principal regulator of the key glycogen metabolism enzymes glycogen synthase (GS) and glycogen phosphorylase (Phosphorylase) in muscle, PP2A is an important regulator of these enzymes in the liver [25, 26]. GS and Phosphorylase are reciprocally regulated. PP2A-mediated dephosphorylation results in activation of GS [27]. Meanwhile, PP2A-mediated dephosphorylation of phosphorylase converts the active, phosphorylated form termed “Phosphorylase a” into the inactive, dephosphorylated form termed “Phosphorylase b” [27]. We therefore investigated the effects PP2A inhibition had on GS and Phosphorylase activity and found that LB1-treatment resulted in GS inactivation and a ~35% increase in hepatic GS phosphorylation (Figure 6a) while leading to an ~45% increase in Phosphorylase a/b ratio (Figure 6b) and thus, Phosphorylase activation. Similarly, ip. injection of cantharidin (0.5mg/kg) resulted in an increase in both GS and Phosphorylase phosphorylation after 30 mins (not shown). *In vitro* PP2A-inhibition in primary hepatocytes mimicked these effects, with 30 mins of cantharidin-treatment resulting in a ~50% increase in the phospho-GS/GS ratio (Figure 6c) and a striking six-fold increase in the Phosphorylase a/b ratio (Figure 6d). PP2A is also known to dephosphorylate and activate the key lipogenic enzyme Acetyl-CoA Carboxylase (ACC) [25] and to dephosphorylate and inactivate the “low-energy” sensor AMP-activated protein kinase (AMPK) [28]. Accordingly, we found that hepatic PP2A-inhibition in rats with LB1 resulted in a ~50% increase in ACC phosphorylation (Figure S5a) and a 2-fold increase in AMPKα phosphorylation (Figure S5b).

### PP2A inhibition leads to impaired insulin-stimulated glycogen synthesis as well as liver glycogen and triglyceride depletion

**Figure 7:**
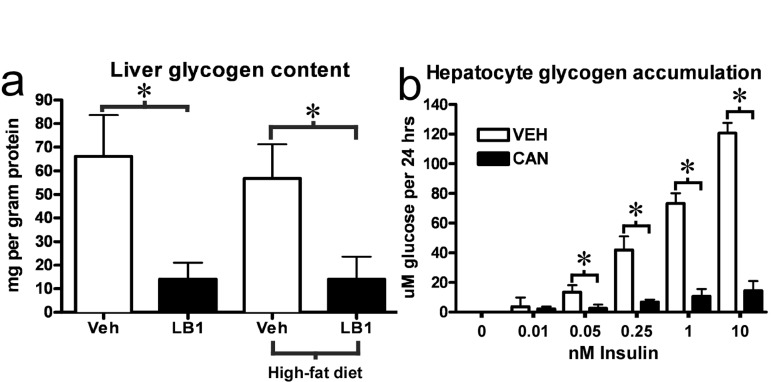
PP2A inhibition by LB1 leads to impaired hepatic insulin-stimulated glycogen synthesis, and, thus, glycogen depletion. Post-clamp glycogen content was significantly reduced in the livers of LB1-treated rats **(a)**. Consistent with this, PP2A inhibition by cantharidin resulted in an impairment of insulin-stimulated glycogen synthesis in primary rat hepatocytes **(b)**. Data are averages ±SEM. * P<0.05.

Our results clearly suggested that PP2A inhibition impaired net glycogen synthesis through impairment of GS activity and glycogen synthesis as well as stimulation of phosphorylase activity and glycogen breakdown. We therefore investigated the livers of the clamped, LB1-treated rats. We found that at the end of the clamp, PP2A inhibition was associated with a marked reduction in the hepatic glycogen content of LB1-treated rats (Figure 7a) (~11 and 12ug/mg protein for chow- and fat-fed respectively) compared to vehicle-treated rats (~65 and 55 ug/mg protein). Again, we found similar effects of PP2A inhibition *in vitro*, as cantharidin-treatment very efficiently impaired insulin-stimulated glycogen accumulation in hepatocytes (Figure 7b). It should be noted, however, that this prolonged cantharidin-treatment was stressful to the hepatocytes and associated with a ~25% increase in cell death (not shown). As our results had indicated PP2A also promotes ACC activation and AMPKα inactivation we examined liver triglyceride levels in rats treated with 2mg/kg LB1 for three days. In line with our findings indicating that PP2A activity promotes nutrient storage, we found that LB1-treatment resulted in a ~50% reduction in fasting hepatic triglyceride levels in chow-fed rats (Figure S6).

## DISCUSSION

In recent years, focus within the field of hepatic insulin action has been predominantly on the effects mediated by Akt, the assumption being that a principal effect of insulin is to activate Akt. Akt then inhibits FoxO1 thus suppressing gluconeogenic gene transcription and gluconeogenesis [5, 29] while simultaneously acting to stimulate net glycogen synthesis through inactivation of Gsk3α [10] – with the combined effect of these actions being a decrease in hepatic glucose production. Recent work by Lu et al. [30] has, however, indicated that insulin-stimulated hepatic Akt activity is dispensable in the absence of FoxO1 while other studies have found that inactivation of Gsk3α plays only a minor role in insulin-stimulated glycogen synthesis [31], the implication being, that other insulin-stimulated signaling pathways are important for acute regulation of hepatic glucose production. We found that PP2A inhibition increased white adipose tissue insulin sensitivity and resulted in a marked enhancement of insulin signaling through the Akt node, but nevertheless exacerbated whole-body and hepatic insulin resistance through an impairment of insulin-stimulated glycogen synthesis. The acute worsening of insulin resistance, despite enhanced Akt phosphorylation and Akt-FoxO1 signaling, highlights that acute regulation of hepatic glucose production by insulin is unlikely to be mediated by transcriptional changes in gluconeogenic enzymes. Rather, our findings suggest that the relative glycogen synthase and phosphorylase activities are important in this context, and that stimulation of net glycogen synthesis is the principal way by which hepatic insulin action acutely regulates plasma glucose levels in the post prandial state. This is compatible with previous studies by Petersen et al. [4] and more recent findings by Ramnanan et al. [32] demonstrating that insulin primarily suppresses hepatic glucose production through modulation of glycogen metabolism and indicating that non-Akt signaling nodes deserve more attention in this process.

PP2A hyperactivity has been implicated in the pathogenesis of insulin resistance and diabetes [33]. While many studies have focused on the inhibitory effects of PP2A on insulin signaling through Akt, PP2A controls the phosphorylation level of a number of proteins and our findings suggest that other hepatic targets are important in the regulation of glucose homeostasis. That inhibition of PP2A resulted in an acute worsening of insulin resistance indicates that PP2A activity is required for insulin-stimulated glycogen storage *in vitro* and *in vivo*. Aside from fatty acids, other nutrients such as ethanol [34] and glucose [35] are known to increase hepatic PP2A activity. PP2A dephosphorylates and activates the key lipogenic enzyme ACC [25] as well as the lipogenic transcription factor carbohydrate responsive element binding protein (ChREBP) [36]. Further, increased PP2A action indirectly leads to intensified activity of several hepatic enzymes and transcription factors that promote energy storage (i.e. GS, ACC, ChREBP and HMG-CoA reductase) [28] as PP2A inhibits AMPK [37] that acts as a “low-energy” sensor and suppresses the activity of these proteins [28].

Genetic knockout approaches of PP2A are problematic, as the exact regulatory subunits that target PP2A to specific substrates remain largely unknown while knockout of the catalytic subunit results in either lethality or no phenotype due to the broad spectrum of importance of PP2A activity and strong compensatory regulation mechanisms, respectively [38]. Due to these concerns, we therefore chose to study the role of PP2A in glucose metabolism using the specific small molecule inhibitors cantharidin and LB1.

The number of substrates controlled by PP2A as well as the many regulatory subunits employed by PP2A complicates its study and further studies are needed to fully elucidate the roles played by the phosphatase. However, in light of the studies, by our and other groups, finding that PP2A activity is stimulated by nutrients to promote glycogen- and lipid storage through acting on key enzymes and transcription factors such as GS, phosphorylase, ACC, AMPK and ChREBP we propose a model where the role of PP2A hyperactivity in states of overnutrition represents a normophysiological adaptation to promote energy storage (Figure S7).

In conclusion, we find that PP2A hyperactivity is not primarily a mediator of the negative effects of lipid overflow on hepatic insulin action, but rather a signal to promote nutrient storage, thus making it unsuitable as a target for therapeutic intervention.

## METHODS

### Animals

All animals were housed at Yale University School of Medicine and maintained in accordance with the Yale University Institutional Animal Care and Use Committee (IACUC) guidelines. All experimental procedures were approved by the Yale University IACUC. Animals were euthanized by pentobarbital sodium infusion.

### Hepatocyte culture

Hepatocytes were isolated by collagenase perfusion as described previously [39] from male Sprague-Dawley rats (200-300g, 8-10 weeks old, Taconic). The hepatocytes were cultured in medium 199, 5.5 mM glucose + GlutaMAX (Gibco) + 0.5% Human Serum Albumin (HSA) (Sigma) + 100nM Decadron (MSD) + 1% Penicillin/Streptomycin (Gibco). For all hepatocyte experiments n=5-6.

### Real-time PCR

RNA was isolated from cells using the RNeasy Mini Kit (Qiagen). cDNA was synthesized using the iScript kit (Bio-Rad). Primer-probesets were from TaqMan Gene Expression Assays (Applied Biosystems) and PCR reactions were performed using a TaqMan Master mix (Applied Biosystem) and a MX3000P system (Agilent). Ct values were normalized to those of Cyclophilin B (Ppib).

### Western Blotting

Protein lysates were assayed with antibodies against IRβ, Akt, pAkt (Ser473), Akt2, FoxO1, pFoxO1 (Ser256), pGS (Ser641), GS, pGsk3α (Ser21), Gsk3α, πAMΠKα (Thr172), AMPKα, pACC (Ser79), ACC, GAPDH (Cell Signaling Technology), pAkt2 (Ser474) (Signalway Antibody), phospho-tyrosine (4G10, Millipore), G6Pase-α, Pepck (Santa Cruz), β-actin (Sigma), phoshorylase a and b (kind gifts from Dr. Brady, University of Chicago). Secondary antibodies were horseradish peroxidase-coupled and ECL reagent (BioVision) was used for detection. Quantification was performed using ImageGuage 4.0 (Fujifilm). Phosphorylation levels of individual proteins were normalized to the total amounts of the respective proteins. Total protein levels were normalized to β-actin or GAPDH. For pFoxO1 and pIRβ blots, FoxO1 or IR was immunoprecipitated before blotting for pFoxO1 or phospho-tyrosine. pFoxO1 and phospho-tyrosine values were then normalized to the total amount of immunoprecipitated FoxO1 or IRβ.

### Hepatocyte gluconeogenesis

Hepatocytes were given medium 199 without glucose (Gibco) supplemented with 2 mM pyruvate (Sigma). After 24 hrs of cantharidin-treatment, glucose contained in the medium was measured in a hexokinase/Glu6PDH assay as previously described [40]. Cells contained only minimal amounts of glycogen before stimulation so any contribution of glycogenolysis was negligible.

### Hepatocyte glycogen accumulation

After 24 hrs of insulin stimulation +10uM cantharidin, cellular glycogen was digested with amyloglucosidase (Sigma) for 3 hrs (37°C). Glucose was measured as described above.

### Insulin infusions

Male Sprague-Dawley rats, 200-300g, 8-10 weeks old, Charles River had venous and arterial catheters implanted and externalized as previously described [41]. Animals were given five days to recover and were then put on either a chow or a saturated-fat based diet for three days. Rats were fasted overnight prior to experiments. Three hours before experiments, the animals were injected i.p. with either LB1 (2mg/kg in saline) or vehicle (saline). For acute insulin signaling experiments, rats were given a primed (200mU/kg) continuous [4mU/(kg-min)] infusion of insulin (Novolin, Novo Nordisk) for 20 mins. For hyperinsulinemic-euglycemic clamp experiments, rats were given a primed, continuous insulin infusion (as above) for two hrs. At the same time a variable infusion of [6,6-^2^H] glucose (25% enriched, 20 g/dL) was used to maintain plasma glucose at 100–110 mg/dL. At the end of the experiments, tissues were harvested *in situ* with aluminium tongs pre-cooled in liquid nitrogen and stored at −80°C. For all experiments n=5-6 rats per group.

### Diets

Saturated-fat based diet (D12492) was from Research Diets, unsaturated-fat based (112245) was from Dyets Inc. and both derived 60% calories from fat. Chow diet was 2018S Harlan laboratories.

### Tissue glycogen content

Protein lysates were spotted onto glass microfiber filters (Whatman) and washed in ice-cold ethanol. Filters were dried overnight, the glycogen was digested with amyloglucosidase (Sigma) for 3 hrs (37°C) and glucose was measured as described above.

### PP2A acitivity

PP2A activity was measured using the PP2A immunoprecipitation phosphatase assay kit (Millipore) according to the manufacturer's instructions.

### Chemicals

Palimtate, oleate, lineoleate and cantharidin (Sigma), S961 (Novo Nordisk) and LB1 was a kind gift from Lixte Biotech.

### Triglyceride assay

Was performed using the Triglyceride-SL kit (Genzyme) according to the manufacturer's instructions.

### Statistical analysis

All values are represented as mean+SEM. An unpaired, two-way Student's t-test was performed to determine difference between vehicle and treated groups. P < 0.05 was considered significant. For multiple comparisons, ANOVA was performed followed by t-test.

## SUPPLEMENTARY FIGURES


