# Supplementary material for: PP2A inhibition results in hepatic insulin resistance despite Akt2 activation
Source: Aging (Albany NY). 2013 Oct 21;5(10):770–81. doi: 10.18632/aging.100611 (PMC3838779; doi:10.18632/aging.100611)
Supplement: Supplementary file 1 [file aging-05-770-s001.pdf]

SUPPLEMENTAL FIGURES

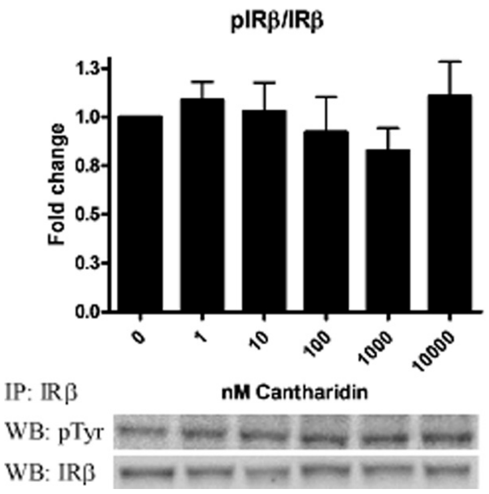

Figure S1. PP2A inhibition does not lead to activation of the insulin receptor. Cantharidin-treatment of primary rat hepatocytes did not result in altered total tyrosine phosphorylation of the insulin receptor. Fold change is relative to no treatment. Data are averages of Western blot quantifications ±SEM. *P<0.05. Representative Western blots are shown.

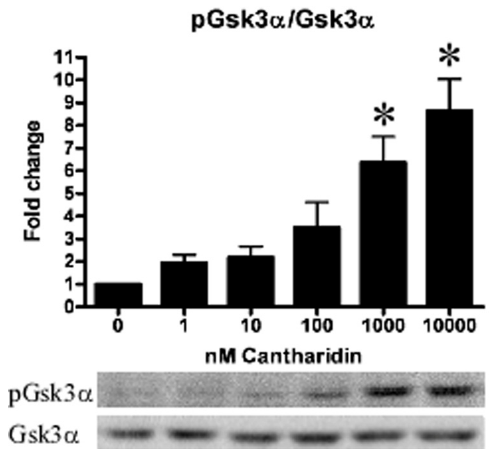

Figure S2. PP2A inhibition results in phosphorylation and inhibition of Gsk3α. In response to cantharidin-treatment, the Akt substrate Gsk3α was phosphorylated (Ser21) and inhibited in primary rat hepatocytes. Fold change is relative to no treatment. Data are averages of Western blot quantifications ±SEM. * P<0.05. Representative Western blots are shown.

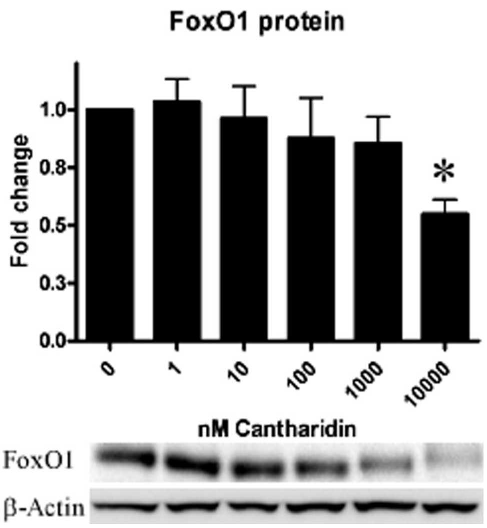

Figure S3. PP2A inhibition results in downregulation of FoxO1. The Akt substrate FoxO1 was downregulated in response to cantharidin in primary rat hepatocytes. Fold change is relative to no treatment. Data are averages of western blot quantifications ±SEM. *P<0.05. Representative Western blots are shown.

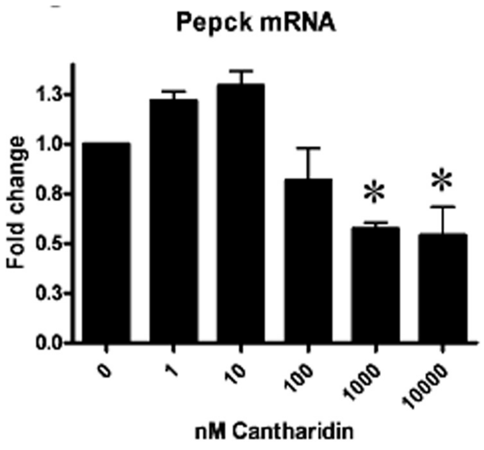

Figure S4. PP2A inhibition leads to downregulation of Pepck mRNA. 2 hrs of cantharidin-treatment resulted in a decrease in transcription of the gluconeogenic enzyme and FoxO1 target gene Pepck. Fold change is relative to no treatment. Data are averages of real-time PCR results ±SEM. *P<0.05.

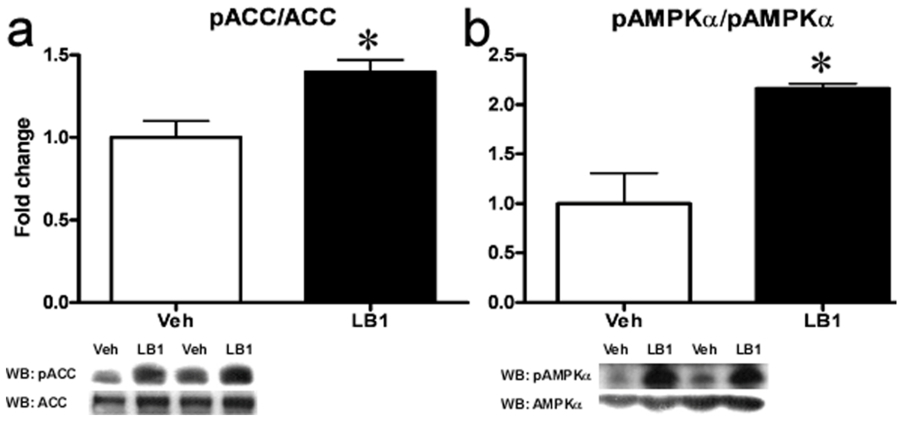

Figure S5. PP2A inhibition results in inactivation of ACC and activation of AMPKα. LB1-treatment resulted in increased phosphorylation (Ser79) and inactivation of ACC (a) while increasing the phosphorylation (Thr172) of AMPKα (b) in the livers of fat-fed rats. Data are averages of western blot quantifications ±SEM. *P<0.05. Representative Western blots are shown.

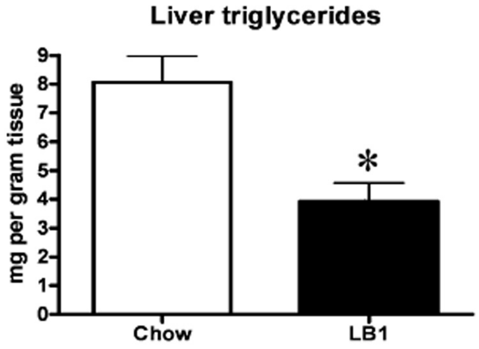

Figure S6. PP2A inhibition by LB1 leads to a lowering in hepatic triglycerides. Liver triglyceride levels were significantly lowered in rats following three days of treatment with the PP2A inhibitor LB1 (a). Data are expressed as averages ±SEM. *P<0.05.

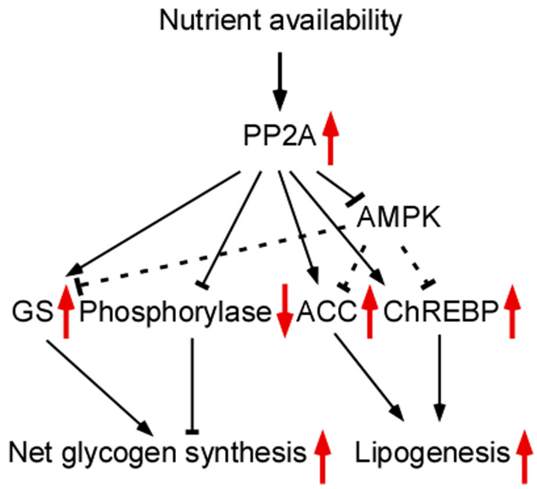

Figure S7. Proposed model for the role of PP2A hyperactivity in states of nutrient availability. Nutrients such as glucose, fatty acids and ethanol induce hepatic PP2A activity. PP2A then promotes energy storage by acting directly on GS and Phosphorylase to stimulate net glycogen synthesis while working to activate ACC and ChREBP to push lipogenesis. Meanwhile, PP2A also stimulates these indirectly through inhibiting AMPK. Black arrows illustrate the direct and indirect (broken arrows) actions of PP2A, while red arrows demonstrate the effects of PP2A hyperactivity.
